# *Cysticercus tenuicollis* in Chamois (*Rupicapra r. rupicapra*) in Germany: Historical Baseline Data, with a Compilation of Records of Taeniid Metacestode Infections in Chamois Throughout Their Range

**DOI:** 10.3390/vetsci13090920

**Published:** 2026-09-07

**Authors:** Dietmar Hamel, Steffen Rehbein

**Affiliations:** Boehringer Ingelheim Vetmedica GmbH, Kathrinenhof Research Center, 83101 Rohrdorf, Germany; dietmar.hamel@boehringer-ingelheim.com

**Keywords:** Alpine chamois, *Rupicapra r. rupicapra*, *Cysticercus tenuicollis*, metacestode of *Taenia hydatigena*, mountain habitats, Germany

## Abstract

Recent publications have indicated an interest in *Cysticercus tenuicollis*, the metacestode (larval stage) of the canid cestode *Taenia hydatigena*, in wild ungulates, but occurrence in mountain ungulates appears to be largely ignored. Therefore, data of chamois from Germany, obtained between 2004 and 2006, were compiled and retrospectively evaluated. *Cysticercus tenuicollis* was found in 27 of 223 (12.1%, CI95 8.3–16.9%) of chamois examined. Infected chamois aged 1 to 15 years and had a low number of one to three cysticerci, which were recovered from abdominal cavity organs exclusively, most often adhering to the mesenteries and the omentum. Variation in the prevalence of infection suggests a positive association of the infection with host age (0% in kids [0/43], 5% in >1-to-3-year-old chamois [5/100], 27.5% in 4-year-old chamois [22/80]; *p* < 0.0001) which was supported by higher *Cysticercus tenuicollis* counts in the ≥4-year-old animals compared to the >1-to-3-year-old animals (0.39 ± 0.74 vs. 0.06 ± 0.28; *p* < 0.0001). Sex appeared to be no risk factor for the occurrence of *Cysticercus tenuicollis* in chamois (prevalence in >1-year-old chamois: 16.2% in males and 13.4% in females). Although considered of minor importance in wild ungulates, *Cysticercus tenuicollis* in chamois is an indicator for the presence of *T. hydatigena* in carnivores thriving or roaming in mountain habitats. To facilitate future research on metacestode infections in chamois, a compilation of the literature on the metacestodes of taeniid cestodes, which have been recorded in both Northern chamois and Southern chamois, is provided.

## Simple Summary

*Cysticercus tenuicollis*, commonly known as the “long-necked bladder worm” or the “thin-necked bladder worm”, is the infective cyst of the tapeworm *Taenia hydatigen*. While *T. hydatigena* is a typical parasite in dogs and also infects other canids, cysts of *Cysticercus tenuicollis* are usually found in the abdominal cavity of a variety of ungulates, mainly ruminants, including both domestic and wild animals. Since the occurrence of *Cysticercus tenuicollis* infection in mountain ungulates seems to have largely gone unnoticed, data collected in the past from the examination of 223 chamois from Germany were compiled and retrospectively evaluated. Cysts of *Cysticercus tenuicollis* were found in 27 (12.1%) of the animals. The cysts, one to three per animal, were attached exclusively to the mesenteries or the omentum. Both the prevalence of infection and the intensity increased with the age of the animals, likely reflecting an age-related cumulative exposure of chamois to the eggs of *T. hydatigena*. Sex did not appear to be a risk factor for the occurrence of *Cysticercus tenuicollis* in chamois. The occurrence of *Cysticercus tenuicollis* in chamois suggests that *T. hydatigena* is present in carnivores that thrive or roam in mountain habitats in Germany.

## 1. Introduction

Ungulates can either be definitive hosts of cestodes, harboring adult tapeworms in their intestinal tract, or they can serve as intermediate hosts in the life cycle of cestodes whose definitive hosts are carnivores, mainly canids, and harbor the infectious larval stages of those cestodes, collectively known as metacestodes. Domestic and wild ruminants are intermediate hosts for the canid-transmitted taeniid cestodes *Taenia hydatigena*, *Taenia multiceps*, *Taenia ovis* and *Echinococcus granulosus* and acquire the infection after accidental ingestion of tapeworm eggs by grazing ground contaminated with the feces of infected dogs or wild canids. The metacestodes of the four cestodes are known as *Cysticercus tenuicollis*, *Coenurus cerebralis*, *Cysticercus ovis* and *Echinococcus hydatidosus*, respectively, and the related parasitic conditions are termed *T. hydatigena* cysticercosis, coenurosis, *T. ovis* cysticercosis and hydatidosis or cystic echinococcosis, respectively. Coenurosis is usually presented clinically, whereas infections with *Cysticercus tenuicollis*, *Cysticercus ovis* and *Echinococcus hydatidosus* are, with rare exceptions of the acute mass infection of migrating *T. hydatigena* cysticerci causing traumatic hepatitis, not clinically evident but may be of relevance at inspection of meat or venison due to condemnation of organs. Importantly, because of the involvement of domestic and wildlife intermediate and definitive host species in the life cycle of these cestodes, domestic, sylvatic and mixed infection cycles may exist [1,2].

Recent publications have indicated an increased interest in *Cysticercus tenuicollis* infection in wild ungulates and *T. hydatigena* infection in canids [3,4,5,6,7,8], but the occurrence of *Cysticercus tenuicollis* in mountain ungulates appears to be largely ignored. This was taken as an opportunity to retrospectively evaluate unpublished data on findings of *Cysticercus tenuicollis* in Alpine chamois from Germany. The evaluation was supplemented by a compilation of the literature on the occurrence of metacestodes in chamois, in order to serve as a basis for further studies on cestode infections in chamois, whose life cycle is essentially based on a predator–prey relationship.

Chamois, represented by the two closely related species Northern Chamois (*Rupicapra rupicapra*) and Southern chamois (*Rupicapra pyrenaica*), with seven and three subspecies, respectively, are the most numerous and widespread mountain ungulates in Europe [9]. A literature search and enquiries made among scientists revealed that metacestodes of four taeniid cestode species have been reported in chamois of seven subspecies (Table 1).

Most information on metacestodes in chamois has been found for the Alpine chamois, the most abundant subspecies of chamois; no such information could be obtained for Chartreuse chamois (*R. rupicapra cartusiana*), Anatolian chamois (*R. rupicapra asiatica*) and Apennine chamois (*R. pyrenaica ornata*). Most publications providing information on metacestodes in chamois report findings of *Cysticercus tenuicollis*, whose occurrence is documented in five subspecies of *R. rupicapra* and two subspecies of *R. pyrenaica*. In contrast, *Coenurus cerebralis* and *Echinococcus hydatidosus* have rarely been reported, merely as sporadic findings. *Coenurus cerebralis* was usually found in chamois presenting clinical signs. One hydatid cyst from an Alpine chamois from France was identified as genotype G2 of *E. granulosus* s.s. [44]. Furthermore, there is one notice on the finding of cysticerci in the heart of two Alpine chamois from the Italian Alps. The cysticerci were named *Cysticercus ovis* in reference to *T. ovis* cysticercosis in sheep and goats [47]. However, since there was apparently no detailed examination of the cysts and no other reports of cysticerci in the muscle tissues of chamois were found, this finding should be treated with caution.

Although *Cysticercus tenuicollis* is arguably the most common metacestode recorded in chamois, there are only a few publications reporting data based on the examination of 100 or more animals [29,35,36,49,105]. Therefore, the findings of *Cysticercus tenuicollis* in 223 Alpine chamois harvested in Germany between 2004 and 2006 were retrospectively evaluated with regard to the relationships between the *Cysticercus tenuicollis* infection and the age and sex of the host animals.

## 2. Materials and Methods

### 2.1. Animals

In a study on the endoparasite status of chamois in Germany, organs of the abdominal cavity and thoracic cavity, which were removed from the carcasses of a total of 223 hunted animals (the offal), were examined [138]. The animals were harvested as part of the regular annual hunting plan in accordance with the German Federal Hunting Act (Bundesjagdgesetz) between September 2004 and December 2006: 185 chamois in the Bavarian Alps, in 13 areas extending over approximately 200 km from the Allgäu Alps in the west to the Berchtesgaden Alps in the east, and 38 chamois in three areas of the Black Forest Mountains. While chamois are native to the Alps in Bavaria, the current population of chamois in the Black Forest Mountains was established through the translocation of chamois from Austria in the 1930s [9,139].

The total sample included 43 kids (‘young of the year’; 19 male, 24 female), 100 chamois aged 1 to 3 years (61 male, 37 female [no information on sex was available for two animals]) and 80 chamois aged 4 to 18 years (50 male, 30 female). Information on the age (determined by horn ring counting) and sex of the animals was provided by the hunters.

### 2.2. Parasitological Methods

The search for *Cysticercus tenuicollis* was carried out by examination of the viscera of all 223 chamois (gastrointestinal tract with spleen, kidneys, bladder and reproductive organs, and liver of 190 animals) and thoracic cavity organs of 203 of those 223 chamois (lungs with mediastinum, and heart of 183 animals) prior to further processing for parasite recovery. The outer surface (serous membranes) of the organs was examined grossly for the presence of cysticerci. When encountered, cysticerci were counted and removed. The preliminary identification was based on the characteristic morphological features of *Cysticercus tenuicollis* (cyst containing a single long-necked inverted scolex in transparent fluid) and confirmed by microscopic examination of the everted scolex using published descriptions of the rostellum hooks (regarding number, shape and size) [140,141].

### 2.3. Data Analysis

Since neither the ratio of the numbers of the chamois in the three age groups (36/79/70 vs. 7/21/10; *p* > 0.1) nor the prevalence of *Cysticercus tenuicollis* (see Section 3) differed statistically between the cohorts originating from the Bavarian Alps and the Black Forest Mountains, the data of all animals were combined for the evaluation of relationships between the prevalence and burden of *Cysticercus tenuicollis* and the age and sex of the chamois. Descriptive statistics and Fisher’s exact tests on proportions were performed in Microsoft^®^ Excel^®^. *Cysticercus tenuicollis* counts were compared using the Mann–Whitney U test.

## 3. Results

In the organs of the 223 chamois examined, a total of 37 mature cysticerci were removed from 27 chamois of 1 year to 15 years of age, giving a sample prevalence of 12.1% (confidence interval [CI95] 8.3–16.9%). The infected chamois harbored one to three *Cysticercus tenuicollis* (median, 1); 20/27 animals had a single cyst (74.1%), two and three cysts, respectively, were recovered from 4/27 (14.8%) and 3/27 (11.1%) animals. The cysticerci presented the characteristic morphology of a thin-walled, transparent, fluid-filled vesicle (‘bladderworm cyst’) with a single inverted protoscolex, and all were identified as *Cysticercus tenuicollis*. Cysticerci were recovered from the abdominal cavity exclusively, adhering to the mesenteries and the omentum; no cysticerci were associated with the liver or spleen. No other metacestodes were encountered.

No statistically significant difference was detected in the prevalence of infection between chamois from the Bavarian Alps and the Black Forest Mountains (10.8% [20/185] vs. 18.4% [7/38]; *p* > 0.1). The prevalence of infection varied between the age groups (0% [0/43] in kids, 5% [5/100] in chamois of >1 to 3 years and 27.5% [22/80] in chamois of ≥4 years; *p* < 0.0001), suggesting a positive association between prevalence and host age. No statistically significant difference was detected in the prevalence of *Cysticercus tenuicollis* infection between male and female chamois over 1 year of age: 16.2% (18/111) vs. 13.4% (9/67), respectively (*p* > 0.1). Among chamois over 1 year of age, the ≥4-year-old animals had more cysticerci than the >1-to-3-year-old animals (0.39 ± 0.74 vs. 0.06 ± 0.28; *p* < 0.0001).

## 4. Discussion

To the authors’ knowledge, the results presented here of the examination of 223 hunter-harvested chamois from Germany, as well as the information on the finding of C. tenuicollis in 3 of 284 chamois from Slovenia (animals found dead or culled in poor condition, necropsied between 2000 to 2020; Vengušt et al. [105] and Žele Vengušt, *in litt*., 28 August 2025) represent the most current data on the occurrence of *Cysticercus tenuicollis* metacestodes in Alpine chamois.

The mean prevalence estimates based on combining data from publications on studies conducted at different times and locations and with different sample structures are subject to limitations and should therefore be interpreted with due caution. Accepting some variability due to the aforementioned potential biases, the compilation of prevalence information, reported in publications with a sample size of at least 10 animals yielded combined samples of 63 to 913 Alpine chamois and estimated mean prevalence values for *Cysticercus tenuicollis* in the range of approximately 1% to 50% per country: France, ~25% (n = 913—[18,25,26,31,34,35,36,38]); Italy, ~49% (n = 131—[49]); Switzerland, ~14% (n = 63—[53,54]); Austria, ~41% (n = 109—[69,88,97]); Czechia, ~14% (n = 148—[113,116]), and Slovenia, ~1% (n = 284—Vengušt et al. [105] and Žele Vengušt, *in litt*., 28 August 2025). The prevalence of *Cysticercus tenuicollis* infection found in Alpine chamois from Germany was therefore in the lower half of the values estimated for Alpine chamois in other European countries. Using the above-mentioned approach to data combination yielded *Cysticercus tenuicollis* prevalence values of ~25% for Cantabrian chamois from Spain (n = 166—[12,14]) and ~60% for Pyrenean chamois from Spain and France (n = 278—[11,13,27,28,29,32]). Information on *Cysticercus tenuicollis* in Tatra chamois, Carpathian chamois, Balkan chamois and Caucasian chamois was insufficient to allow for an estimation of the prevalence of the infection.

The differences in the prevalence of *Cysticercus tenuicollis* infections between the various chamois populations could reflect the varying frequency of *T. hydatigena* in the potential carnivore hosts capable of contaminating the chamois’ habitat with their feces. For chamois in Germany, red fox (*Vulpes vulpes*) and dogs, mainly hunting dogs, may constitute relevant final hosts of *T. hydatigena*. Foxes may acquire *T. hydatigena* by scavenging on chamois (e.g., winter deaths) [142,143,144], and feeding on the raw organs or offal of chamois or other ungulates can be a source of infection for hunting dogs. To the authors’ knowledge, no data are available on *Taenia* cestodes in foxes and dogs from the regions of the Bavarian Alps and Black Forest Mountains in Germany. However, *T. hydatigena* has been identified in foxes from the Italian Alps [3], and recently, wolves from the Alps in Italy, France and Switzerland were found infected with *T. hydatigena* to a considerable extent [4,145,146,147]. One other potential source of infection in chamois may be dogs that are used for herding and protecting livestock on alpine pastures [145]; however, this husbandry system is not practiced in Germany.

The number of *Cysticercus tenuicollis* per chamois in the present investigation was low und thus in line with information provided in most papers on *T. hydatigena* metacestodes in chamois. The maximum counts reported in chamois were 12, 7 and 6 *Cysticercus tenuicollis* cysts, recorded in single Carpathian chamois, Tatra chamois and Pyrenean chamois, respectively [29,118,123]. Neither our own investigation nor the reviewed literature have provided any evidence of impact of the infection in chamois.

The finding that the cysticerci were adhered to the mesenteries and omentum in the chamois from Germany was generally in agreement with all publications that provided information on the localization of *Cysticercus tenuicollis* cysts in chamois. However, there are also reports of *Cysticercus tenuicollis* being adhered to the liver [11,14,17,18,19,20,24,36,37,49,71,75,77,90,93,94,115,123,127], the spleen [94] and the lungs [11,74,94,115] or on the pericardium [49,50] in chamois. The organ localization pattern of *Cysticercus tenuicollis* cysts in chamois thus corresponds to that observed in sheep, with the mesentery and omentum being the preferred predilection sites [148].

The prevalence and number of *Cysticercus tenuicollis* in the Alpine chamois from Germany suggest that older animals are more frequently infected and have more cysts than younger ones. This is supported by the evaluation of information on a total of 119 Pyrenean chamois when combining the data reported by Alcouffe [29] and Gonzales Iglesia [11]: both, *Cysticercus tenuicollis* prevalence (18.8% [3/16] in kids, 52.5% [21/40] in chamois of 1 to 3 years of age and 60.3% [38/63] in chamois of ≥4 years of age; *p* = 0.0122) and *Cysticercus tenuicollis* numbers (0.31 ± 0.79 in kids, 0.85 ± 0.95 in 1-to-3-year-old animals and 1.30 ± 1.53 in ≥4-year-old animals; *p* = 0.012) were positively associated with the age of the chamois.

The gradual increase in the prevalence and number of cysts of *Cysticercus tenuicollis* with the increasing age of the animals probably reflects an age- and diet-related cumulative exposure to tapeworm eggs.

In line with findings in the chamois from Germany, the analysis of data from >1-year-old male and female Pyrenean chamois [29] revealed no statistical difference in the prevalence of *Cysticercus tenuicollis* infection between male and female animals (62.2% [23/37] vs. 42.0% [21/50], *p* = 0.0834).

## 5. Conclusions

The results of the retrospective evaluation of data on the occurrence of *Cysticercus tenuicollis* in chamois from and the review of the literature indicate that *Cysticercus tenuicollis*, the metacestode of the tapeworm *T. hydatigena*, is a common parasite of chamois, the most abundant mountain ungulates in Europe. This work provides historical baseline information for further studies into the distribution of *T. hydatigena* metacestode infections in mountain ungulate habitats in Europe. Because both metacestode and adult cestode infect domestic and wild animals, the parasite may be transmitted in sylvatic, domestic and mixed domestic/sylvatic cycles and is thus worthy of study with chamois serving as sentinels. Specifically, further studies are desired to identify the final hosts of *T. hydatigena* in the habitats of mountain ungulates.

## Figures and Tables

**Table 1:** *Cysticercus tenuicollis* (Ct), *Cysticercus ovis* (Co), *Coenurus cerebralis* (Cc) and *Echinococcus hydatidosus* (Eh) records of natural infections in chamois throughout their range.

	Northern Chamois— *Rupicapra rupicapra*					Southern Chamois – *Rupicapra pyrenaica*	
	Alpine chamois (*R. r. rupicapra*)	Tatra chamois (*R. r. tatrica*)	Carpathian chamois (*R. r. capaticaa*)	Balkan chamois (*R. r. balcanica)*	Caucasian chamois (*R. r. caucasica*)	Pyrenean chamois (*R. p. pyrenaica*)	Cantabrian chamois (*R. p. parva*)
**Spain ^1^**						**Ct, Cc, Eh**	**Ct, Eh**
**Andorra ^2^**						**Ct**	
**France ^3^**	**Ct, Cc, Eh**					**Ct, Eh**	
**Italy ^4^**	**Ct, Cc, Co?**						
**Switzerland ^5^**	**Ct, Cc, Eh**						
**Germany ^6^**	**Ct, Cc**						
**Austria ^7^**	**Ct, Cc, Eh**						
**Slovenia ^8^**	**Ct**						
**Croatia ^9^**	**Ct**						
**Bosnia and Herzegovina ^10^**				**Ct, Cc**			
**Bulgaria ^11^**				**Eh**			
**Czechia (Czech part of former Czechoslovakia and current Czechia) ^12^**	**Ct, Eh**						
**Slovakia (Slovak part of former** **Czechoslovakia and current Slovakia) ^13^**	**Ct**	**Ct, Cc**					
**Romania ^14^**			**Ct, Cc, Eh**				
**Russia (Caucasus Nature Reserve,** **Kabardino-Balkaria, Dagestan) ^15^**					**Ct, Eh**		
**Georgia ^16^**					**Ct, Eh**		
**Azerbaijan ^17^**					**Ct, Eh**		

^1^ **Ct** [10,11], **Ct** + **Eh** [12,13,14], **Cc** [15,16]; ^2^ **Ct** [17]; ^3^ **Ct** [18,19,20,21,22,23,24,25,26,27,28,29,30,31,32,33], **Ct** + **Cc** [34,35,36,37], **Ct** + **Cc** + **Eh** [38], **Cc** [39,40,41], **Eh** [42,43,44]; ^4^ **Ct** [45,46], **Ct** + **Cc** [47,48], **Cc** [49], **Co?** [50]; ^5^ **Ct** [51,52,53], **Ct** + **Eh** [54], **Cc** [55,56,57]; ^6^ **Ct** [58], **Cc** [59,60,61]; ^7^ **Ct** [62,63,64,65,66,67,68,69,70,71,72,73,74,75,76,77,78,79,80,81,82,83,84,85,86,87,88], **Ct** + **Eh** [89,90,91,92,93,94,95,96,97], **Cc** [61,98,99,100,101], **Cc** + **Eh** [102], **Eh** [103,104]; ^8^ **Ct** [Vengušt et al. [105] and Žele Vengušt, *in litt*., 28 August 2025]; ^9^ **Ct** [106,107]; ^10^ **Ct** + **Cc** [108], **Cc** [109]; ^11^ **Eh** [110]; ^12^ **Ct** [111,112,113,114], **Ct** + **Eh** [115,116,117]; ^13^ **Ct** [7,118,119,120,121], **Cc** [122]; ^14^ **Ct** [123,124], **Cc** [125], **Ct** + **Cc** + **Eh** [126]; ^15^ **Ct** [127,128,129], **Ct** + **Eh** [130], **Eh** [131,132,133,134]; ^16^ **Ct** [135], **Ct** + **Eh** [136]; ^17^ **Ct** [137], **Eh** [131].

## Data Availability

The original contributions presented in this study are included in the article. Further inquiries can be directed to the corresponding author.
